# Rapid modeling of 3D rice canopy structure considering vertical heterogeneity and analysis of spectral response

**DOI:** 10.1016/j.plaphe.2026.100226

**Published:** 2026-05-25

**Authors:** Juchi Bai, Youwen Tian, Yinxuan Hui, Rui Wu, Fenghua Yu, Chunshan Wang

**Affiliations:** a College of Information and Electrical Engineering, Shenyang Agricultural University, Shenyang, 110866, China; b Smart Agriculture Laboratory in Liaoning Province, Shenyang, 110866, China; c School of Information Science and Technology, Hebei Agricultural University, Baoding, 071001, China

**Keywords:** Rice, 3D radiative transfer model, Vertical heterogeneity, Canopy spectra, Machine learning

## Abstract

The vertical heterogeneity of rice canopy structure limits the accuracy of inverting leaf physicochemical parameters using traditional radiative transfer models, while LiDAR-based 3D reconstruction remains costly for large-scale applications. To address these challenges, this study proposes a method for constructing 3D rice canopy scenes using “Precision Mode” and “Rapid Mode” strategies. The Precision Mode builds detailed structural models based on measured morphological parameters, validated via the LESS 3D radiative transfer model. To overcome the limitations of obtaining detailed morphology via UAV remote sensing, the Rapid Mode employs machine learning algorithms—specifically Support Vector Machine (SVM), Random Forest (RF), and XGBoost—to map easily accessible parameters (LAI, Above-ground Biomass, Plant Height, and Transplanting Date) to detailed 3D structural parameters. Results indicate that XGBoost achieves the highest accuracy in the Rapid Mode. Furthermore, simulated spectra under both modes showed high consistency with measured spectra, yielding average RMSE values of 0.0104 (R^2^ = 0.9965) for the Precision Mode and 0.0307 (R^2^ = 0.9694) for the Rapid Mode. Although the spectral accuracy of the Rapid Mode is slightly lower, its modeling efficiency is significantly enhanced, retaining a strong capability to reproduce spectral response characteristics across growth stages. This approach provides an effective tool for analyzing vertical spectral response mechanisms and offers an efficient data simulation scheme for UAV remote sensing parameter inversion based on 3D radiative transfer models.

## 1 Introduction

He chlorophyll and nitrogen contents of rice leaves significantly characterize the nutritional status and photosynthetic efficiency of rice; therefore, leaf-level nutritional diagnosis is of great significance for rice fertilization management and yield estimation [1,2]. According to the critical nitrogen dilution theory, under conditions of sufficient nutrient supply, nitrogen absorbed by roots is preferentially allocated to the upper leaves, making them less sensitive to variations in nitrogen fertilizer application, whereas the nitrogen status of middle and lower leaves more accurately reflects the actual nutritional status of the rice plant [3]. Research on vertical nitrogen distribution also indicates that under different nitrogen treatments, there are significant differences in nitrogen accumulation along the vertical direction of the canopy, which are closely related to yield [4]. However, due to the shading effect of upper leaves, the spectral characteristics of middle and lower leaves are less distinct than those of the upper layers. This is a major source of error in hyperspectral-based rice nutritional diagnosis methods [5]. Therefore, elucidating the coupling mechanism between rice canopy vertical structure and leaf spectra is crucial for rice nutritional diagnosis and photosynthetic efficiency estimation.

As a key tool for explaining the coupling mechanism between crop canopy structure and leaf spectra, the canopy radiative transfer model provides a physical explanation of the crop canopy radiative transfer process based on canopy assumptions, combined with leaf spectra and radiative transfer solution methods [6]. Depending on different canopy assumptions, canopy radiative transfer models are categorized into one-dimensional radiative transfer models, geometric optical models, and 3D radiative transfer models. Among them, one-dimensional radiative transfer models (such as PROSAIL) typically assume the canopy as a turbid homogeneous medium. This assumption makes it difficult to reflect the vertical heterogeneity of the rice canopy [7]. Although some models, such as the SPCOE model, attempt to describe canopy vertical heterogeneity by dividing the canopy into multiple heterogeneous layers, this heterogeneity reflects macroscopic laws at the vertical scale and does not link specific leaf structures to spectra [8]. Geometric optical models explain the canopy radiative transfer process by assuming the canopy as simple geometric bodies, which similarly struggle to describe the impact of rice canopy vertical heterogeneity on spectra [9]. Three-dimensional radiative transfer models (such as LESS, DART) construct canopy structures that are close to reality through computer modeling methods and simulate the canopy radiative transfer process combined with ray tracing or discrete ordinate methods [10,11] Since 3D radiative transfer models can input fine-scale 3D structures and assign different optical properties to different structures, they can effectively explain the coupling mechanism between rice canopy vertical structure and leaf spectra, making them an effective method for studying vertically heterogeneous canopy structures.

The explanatory capability of 3D radiative transfer models regarding canopy structure depends on the refinement level of the 3D scene construction. Currently, relevant research mainly utilizes Functional-Structural Plant Models (FSPM) to simulate crop 3D structures. FSPM simulates the plant growth and development process based on physiological functions, thereby reproducing the plant's 3D structure. Commonly used FSPMs include GreenLab, GroIMP, etc. [12,13]. These models can simulate rice 3D structures based on inputs of actual parameters such as leaf length and leaf width. Qian combined a maize functional-structural model with the MAIZSIM crop growth model to construct a 4D maize growth model capable of simulating temporal changes in maize 3D structure [14]. Chang simulated the effects of rice canopy structure and vertical leaf nitrogen content on canopy radiation use efficiency by constructing a rice FSPM [15]. However, these models primarily focus on simulating rice phenotypic characteristics and growth processes, without establishing a direct connection with radiative transfer models. Moreover, the inputs required by these models, particularly vertical-scale structural parameters such as leaf length and leaf width, are challenging to obtain rapidly using UAV remote sensing techniques. Although terrestrial point cloud-based and multi-view RGB photographic methods can provide detailed rice point cloud data and detect these parameters, their high measurement costs make them unsuitable for large-scale 3D scene reconstruction. Although numerous studies have demonstrated that UAV remote sensing methods can quantitatively detect canopy structural parameters such as LAI and Above-ground Biomass (AGB)—for instance, Zhang et al. attempted to estimate rice LAI using UAV RGB and multispectral data [16]—the limited accuracy of remote sensing data and occlusion effects within rice canopies make the detection of vertical leaf structural parameters extremely difficult. Given that many FSPMs and rice structural studies have indicated that canopy structural parameters are potentially correlated with vertical leaf structural parameters considering the growth characteristics of rice [17,18], obtaining canopy structural parameters through multispectral and LiDAR methods, and then constructing a mathematical relationship between canopy and vertical leaf structural parameters using in-situ measurements, could enable indirect rapid estimation of vertical leaf structural parameters, thereby facilitating rapid simulation of rice field 3D scenes.

Currently, there is still a lack of methods that can rapidly construct 3D structures capable of reflecting the influence of rice leaf vertical heterogeneity on canopy spectra. This implies that a 3D structural model should meet the requirement of accurately simulating the relationship between “vertical leaf reflectance and canopy reflectance.” It should be able to achieve precise simulation of the rice 3D structure by obtaining specific 3D structural parameters required by the model through actual measurements, and also achieve rapid simulation of the rice 3D structure by inputting canopy structural parameters that can be rapidly acquired via remote sensing methods to derive high-precision vertical leaf structural parameters through inversion modeling methods. Therefore, the rice 3D structural model constructed in this study includes a “Precision Mode” that simulates 3D structure by inputting vertical leaf structural parameters, and a “Rapid Mode” that simulates using canopy structural parameters such as LAI, AGB, and Plant Height (PH) that can be estimated by remote sensing methods. This allows the model to be used not only to analyze the impact of canopy vertical structural parameters and leaf spectral changes on canopy spectra but also to be applied to the rapid reconstruction of rice field 3D scenes and canopy radiation use efficiency simulation. At the same time, it can also be coupled with crop growth models to simulate the rice growth process.

## 2 Materials and methods

### 2.1 Experimental design

**Fig. 1:**
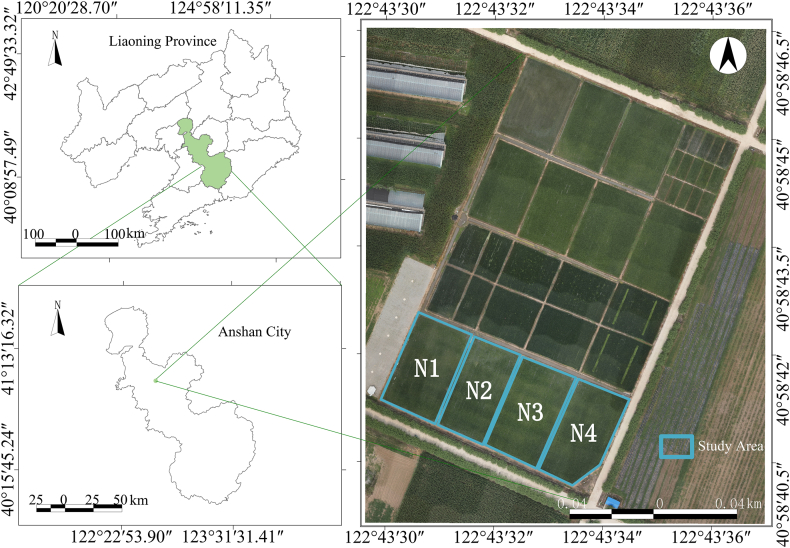
Distribution of the experimental area.

A field experiment was conducted from June to August 2024 at the Precision Agriculture Aviation Research Base of Shenyang Agricultural University, located in Gengzhuang Town, Haicheng City, Anshan City, Liaoning Province (40°58′45.39″ N, 122°43′47.0064″ E). Four nitrogen fertilizer treatments were established: N1 = 165.3750 kg ha^−1^, N2 = 110.2500 kg ha^−1^, N3 = 275.6250 kg ha^−1^, and N4 = 220.5000 kg ha^−1^. All other field management practices were held consistent across treatments, with the specific layout illustrated in Fig. 1. Field sampling was conducted from the tillering to booting stage, with sampling dates and details provided in Table S6. During each sampling event, four random locations were selected within each plot, from which one representative rice hill was chosen for measurements of canopy hyperspectral reflectance, vertical leaf structural parameters, and leaf-level hyperspectral data. After preliminary screening, a total of 100 samples were selected for subsequent analysis.

### 2.2 Data acquisition and processing methods

#### 2.2.1 Acquisition method of rice 3D structural parameters

We mainly collected structural parameters such as rice AGB, LAI, leaf length, leaf width, leaf area, leaf inclination angle, leaf position, PH, and stem height. The specific sampling plan is as follows: We collected rice LAI data outdoors in the region of interest using an LAI-2200C plant canopy analyzer (LI-COR Inc., Lincoln, NE, USA), and measured rice PH using a ruler; then, the selected one hill of rice was dug out, put into a plastic sealed bag, and brought to the laboratory for indoor experiments. Considering that usually 1 hill of rice contains 10-30 tillers with small differences in growth, the workload of measuring all of them is large, so we selected 3 representative tillers for specific structural parameter measurements, and calculated their average as the structural parameters of this hill of rice. Subsequently, we used a ruler to measure the plant height, stem height, leaf length, leaf width, leaf position, and leaf inclination angle for each rice plant. Specifically, the ruler was used to accurately measure the plant height and stem height, as well as the length and maximum width of each fully expanded leaf. Leaf positions were recorded from bottom to top as L1, L2, and so on, with the distance from the base of each leaf to the rice root base measured as the leaf position. Leaf inclination angle was defined as the angle between the line connecting the leaf sheath and leaf tip and the vertical direction of the stem, measured using a protractor with the leaf in its natural extended state to ensure the data reflected its actual spatial orientation. Leaf area was calculated using formula (1):

(1) $$\begin{array}{c} LA=a*b*0.7746 \end{array}$$

where a is the maximum leaf length, b is the maximum leaf width, and 0.7746 is the leaf area correction coefficient for rice leaves [19].

After acquiring the leaf hyperspectral data, all leaves and stems from one hill of rice were separated and placed into an oven. They were subjected to de-enzyming at 105 °C for 30 min, followed by drying at 70 °C to a constant weight. The dry weights of leaves and stems were measured separately, and their sum was calculated to obtain the AGB.

#### 2.2.2 Acquisition method of rice leaf physicochemical parameters

We measured leaf chlorophyll content (Cab), carotenoid content (Car), Leaf Dry Matter Content (*Cm*), and Equivalent Water Thickness (Cw) in the laboratory for the simulation of rice leaf spectra. After measuring the leaf area and fresh weight of each leaf sample, the Cab and carotenoid content Car were determined. Cab and Car were measured using a spectrophotometer (UV8000PC, Shanghai Macylab Instrument Co. Ltd., Shanghai, China). First, 0.1 g of rice leaf samples were weighed, cut into pieces, and soaked in a 95% ethanol solution in the dark for 72 h. The absorbance of the extract at 649 nm and 665 nm was measured using the UV8000PC. The calculation formulas for Chlorophyll *a* (Ca) and Chlorophyll *b* (Cb) are shown in Equations (2) and (3), and the calculation formula for Car is shown in Equation (4). Finally, the units were converted to $\mu g\cdot {\text{cm}}^{-2}$ [20]:

(2) $$\begin{array}{c} Ca=13.95\;*\;A_{665}-6.88\;*\;A_{649} \end{array}$$

(3) $$\begin{array}{c} Cb=24.96\;*\;A_{649}-7.32\;*\;A_{665} \end{array}$$

(4) $$\begin{array}{c} Car=\frac{1000*A_{470}-2.05*Ca-114.8*Cb}{245} \end{array}$$

where Ca, Cb, and Car represent the contents (%) of chlorophyll *a*, chlorophyll *b*, and carotenoids, respectively; and $A_{470}$, $A_{649}$, and $A_{665}$ represent the absorbance coefficients at 470 nm, 649 nm, and 665 nm, respectively.

Subsequently, the remaining leaf samples were de-enzymed at 105 °C for 30 min and then dried at 70 °C to a constant weight to measure the *Cm* (g) [21]. Finally, the $C_{w}$ ($g\cdot {\text{cm}}^{-2}$) and *Cm* (, $g\cdot {\text{cm}}^{-2}$)were calculated using Equations (5) and (6):

(5) $$\begin{array}{c} C_{w}=\frac{\left(\text{FW}-\text{DW}\right)}{\text{LA}} \end{array}$$

(6) $$\begin{array}{c} C_{m}=\frac{DW}{LA} \end{array}$$

FW stands for leaf fresh weight; DM stands for leaf dry weight; LA stands for rice leaf area.

#### 2.2.3 Acquisition method of rice canopy spectral data

This study employed a portable spectroradiometer (ASD FieldSpec HandHeld 2, Analytical Spectral Devices, Boulder, CO, USA) to acquire hyperspectral reflectance data of the rice canopy. The instrument possesses a spectral detection range of 325–1075 nm, a spectral resolution of 3 nm, a sampling interval of 1.5 nm, and a Field of View (FOV) of 25°. Spectral acquisition was conducted during periods of stable illumination (10:00–14:00 Beijing Time). During measurements, operators wore dark clothing and faced away from the sun. The sensor probe was positioned vertically approximately 1.0 m above the rice canopy to ensure that the field of view covered a representative rice population while avoiding interference from shadow projection. Radiometric calibration using a standard white reference panel was performed before and after each measurement to eliminate the influence of varying illumination conditions.

#### 2.2.4 Simulation method of rice leaf spectral data

The PROSPECT-5 model was utilized to simulate rice leaf spectra. PROSPECT-5 is a currently widely used Radiative Transfer Model (RTM) capable of simulating leaf reflectance and transmittance based on input parameters. The model incorporates six input parameters: structural parameter (N, Cab, Cw, *Cm*, Car, and brown pigment [Cbrown]). By running the PROSPECT model in forward mode, directional-hemispherical leaf reflectance and transmittance can be generated within the 400–2500 nm spectral range at a 1 nm resolution. In this study, leaf spectra were simulated based on measured leaf physicochemical parameters, with N set to 1.5 and Cbrown set to 0. Consistent with the canopy spectral range, the simulated leaf spectra were also limited to the 400–1000 nm waveband.

### 2.3 Construction method of rice 3D structural model under “Precision Mode"

To achieve refined modeling of the canopy structure for individual rice plants and single hills, this study designed a 3D geometric construction workflow based on parametric modeling methods, modeling the 3D structures of leaves, stems, and single rice hills, respectively. Implemented in the Python programming language, the model integrates geometric modeling algorithms with procedural random perturbation strategies to generate visualized 3D canopy models with biological realism.

#### 2.3.1 3D geometric modeling of leaves

In this study, the 3D geometric structure of leaves is simulated via parametric modeling. The model abstracts the leaf as a set of points generated along the midrib direction. And the morphological changes in the vertical direction were controlled through functions, thereby achieving simulation of natural leaf curvature and width distribution.

Specifically, the leaf length is determined by the parameter leaf_length, within which discrete points are uniformly sampled at a specified resolution to generate the point set. At each sampling point along the midrib, the transverse width is controlled by a Gaussian-like distribution function based on the normalized position, as expressed below:

(7) $$\begin{array}{c} w\left(z\right)=leaf_{width}*\left[1-{\left(2*\left(\frac{z}{leaf_{length}}-0.5\right)\right)}^{2}\right] \end{array}$$

where W(z) represents the leaf width at position z from the leaf base; $leaf_{width}$ represents the maximum leaf width (usually occurring at the middle of the leaf); $leaf_{length}$ represents the leaf length; and z is the positional variable along the leaf length direction, with a range of $\left[0,leaf_{length}\right]$. This function causes the leaf to narrow gradually at the base and tip while reaching its maximum width in the middle, conforming to the natural morphology of rice leaves.

At the same time, the drooping curvature of leaves in the vertical direction is represented by a quadratic function of z:

(8) $$\begin{array}{c} y\left(z\right)=-k*\frac{z^{2}}{leaf_{length}} \end{array}$$

where y(z) represents the vertical displacement at position z, used to simulate the drooping effect caused by gravity; k is the curvature factor, used to regulate the degree of leaf drooping; and z is the positional variable along the leaf length direction.

This function simulates the natural drooping state of the leaf under gravity. Subsequently, by applying an inclination angle to rotate this curve around the petiole axis, the control of the overall leaf posture is achieved. The inclination angle is applied as Euler angles to the rotation transformation within the z–y plane, thereby generating a realistic 3D spatial curved form.

Furthermore, to enhance the flexibility of the 3D layout, the model allows the leaf to rotate at an arbitrary angle around the plant stem, controlling its offset position in 3D space.

Finally, a pair of vertices is generated at the left and right edges of each sampling point to form a complete leaf cross-section, which is ultimately output as a sequence of 3D coordinate points representing the 3D geometric contour of the leaf edge.

#### 2.3.2 3D structural modeling of stems

The rice stem is abstracted as a cylindrical structure with segment-wise variations, divided into several equal-height sections along the main axis. Each section is a perfect circle divided by equal angles, with its radius decreasing linearly from the base to the top. Vertices are generated via polar coordinates to construct a continuous triangular mesh, realizing the 3D structural modeling of the stem.

#### 2.3.3 Construction of 3D rice model at single-plant scale

In the 3D modeling of a single rice plant, the assembly of leaves and stems is achieved through parameterized spatial transformations. Each leaf is defined by a quadruple parameter containing vertical position, azimuth angle, opening angle, and phyllotaxy index to determine its spatial location. During modeling, the leaf geometry is first generated in a local coordinate system and then accurately attached to the corresponding position on the stem through transformations such as rotation, rotation around the stem, and translation. Finally, all leaves and internodes are integrated to complete a single-plant rice model with a natural spatial structure.

#### 2.3.4 Construction of 3D rice model at single-hill scale

On this basis, the 3D rice structural model at the single-hill scale is composed of multiple single-plant models to simulate the natural field planting pattern. Specifically, multiple plants are distributed in concentric circles, with the number of plants varying across layers. Random perturbations are introduced to the position and rotation angle to enhance the diversity and realism of the population structure. After generation, each plant undergoes the following geometric transformations to integrate into the overall structure: Spatial Translation: Determine its position based on the radius and angular offset of the ring it is located in; Random Rotation: Introduce a rotation angle around the stem to simulate the random growth direction in the field; Tilt Perturbation: Add a random tilting mechanism to the stem to simulate stem tilting and canopy asymmetry.

Finally, the vertex and face information of all plants is unified and written into the.obj file format, which is used for 3D visualization or subsequent applications such as radiative transfer simulation and structural feature extraction. The model output includes the complete model of the entire hill, the separately extracted stem structure, and data for each leaf layer, providing support for multi-scale structural analysis.

### 2.4 Construction method of rice 3D structural model under “"Rapid Mode"

Compared to the “Precision Mode,” the “Rapid Mode” of the rice 3D structural model additionally incorporates a rice vertical leaf structural parameter estimation model. This model estimates vertical leaf structural parameters by inputting canopy structural parameters that can be rapidly acquired via remote sensing platforms, thereby constructing a rice 3D structural model close to the real structure. While the “Precision Mode” requires field measurements of vertical leaf structural parameters or extraction using terrestrial point cloud data, the “Rapid Mode” can utilize remote sensing data acquired by UAVs or satellite platforms to achieve rapid reconstruction of the rice 3D structure, greatly enhancing the model's practicality.

Regarding the rice vertical leaf structural parameter estimation model, this study selected input parameters capable of characterizing rice vertical leaf structure based on the correlation analysis between rice canopy structural parameters and vertical leaf structural parameters, as well as the current status of research on estimating rice canopy structural parameters based on remote sensing methods. Machine learning methods were employed to estimate the vertical leaf structural parameters. Ultimately, the estimation results can be combined with the rice 3D structural model to achieve the rapid generation of the 3D structure of a single hill of rice based on canopy structural parameters.

#### 2.4.1 Correlation analysis of rice structural parameters

**Fig. 2:**
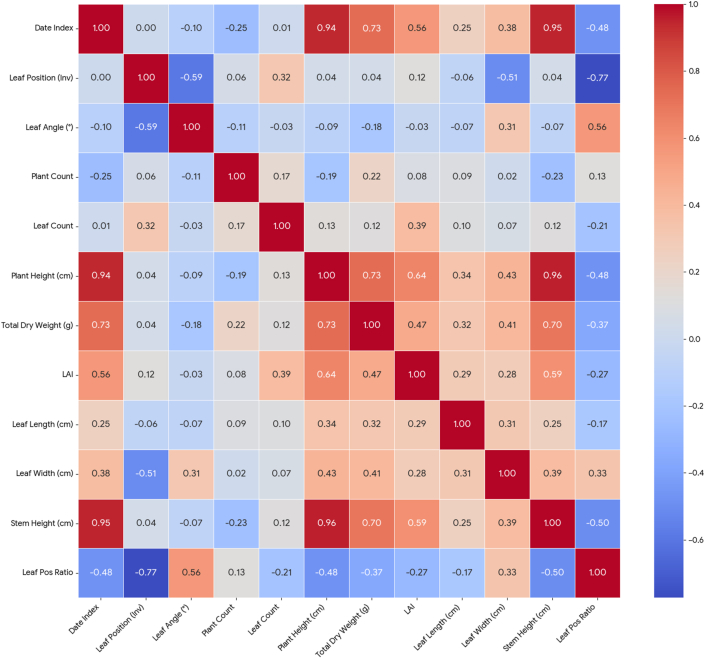
Results of correlation analysis of rice structural parameters.

In this study, Pearson correlation analysis was used to analyze the correlation between rice canopy structural parameters and vertical leaf structural parameters. The analysis results are shown in Fig. 2. There are good correlations among the five parameters: transplanting date, PH, AGB, LAI, and stem height. Among them, the correlations between transplanting date, PH, stem height, and AGB are very strong, while the correlation between LAI and the other four parameters is relatively low. This may be because PH, stem height, and AGB are strongly related to the rice growth stage, while LAI is also influenced by complex factors such as leaf development, canopy structure, and planting density. The correlation between tiller number and other parameters is small, which may be caused by the unfixed number of tillers per hill during transplanting.

Regarding vertical leaf structural parameters, leaf length is correlated with transplanting date, PH, AGB, LAI, and leaf area. Leaf width, leaf position ratio, and leaf inclination angle are all related to leaf position. Combining the laws of rice leaf shape, it can be seen that leaf length may be related to rice development status, while leaf width, leaf position ratio, and leaf inclination angle are also related to the leaf position of the specific leaf, which may be related to the erectophile leaf angle distribution of rice. In general, rice canopy structural parameters such as transplanting date, PH, AGB, and LAI have a certain correlation with vertical leaf structural parameters, indicating that estimating rice vertical leaf structural parameters using canopy structural parameters is feasible.

#### 2.4.2 Estimation method of rice vertical leaf structural parameters

Based on the correlation analysis results from Section 2.4.1 and the difficulty of parameter acquisition, this study employs four parameters—transplanting date, PH, AGB, and LAI—as model inputs. Three machine learning methods, including Support Vector Machine (SVM), XGBoost, and Random Forest (RF), are utilized to estimate canopy structural parameters and vertical leaf structural parameters, with the optimal estimation model selected by comparing estimation accuracy to construct a method for estimating rice vertical leaf structural parameters. To ensure the reliability of model evaluation results, a stratified random splitting strategy is adopted, dividing the original dataset into training and testing sets at a 7:3 ratio. During model training, 5-fold cross-validation is performed on the training set to avoid evaluation bias caused by single splitting. Data normalization is applied, and actual leaf count is used as input when estimating layered leaf structural parameters.

SVM achieves classification and regression by constructing an optimal hyperplane in high-dimensional feature spaces, demonstrating strong generalization capability, particularly for small sample sizes with high dimensionality. XGBoost is an ensemble learning method based on gradient boosting that achieves high-accuracy predictions through weighted integration of multiple weak classification trees, showing excellent performance in handling nonlinear features and large-scale data [22]. RF generates multiple decision trees through random sampling and feature selection, providing final results via voting, which offers good stability and anti-overfitting capability. Regularization is applied to all three models to mitigate overfitting [23].

#### 2.4.3 Calculation method of rice leaf inclination angle function

For the rice leaf inclination angle function, using the average leaf inclination angle data of different leaf positions, the erectophile leaf inclination angle distribution function was adopted to calculate the leaf inclination angle for each leaf position. The leaf inclination angle distribution function was fitted according to the average leaf inclination angle of each leaf position. The fitting results are shown in Fig. S1. It can be seen that the fitting effect of the average rice leaf inclination angle is good (R^2^ = 0.9789,RMSE = 1.4059). The leaf inclination angle function is shown in Equation (9):

(9) $$\begin{array}{c} LIA=85.64-4.12\times LP \end{array}$$

LIA stands for leaf inclination angle; LP stands for leaf position.

### 2.5 Rice 3D scene construction method

The primary method for constructing the rice 3D scene is as follows: First, the corresponding rice 3D structural model is built based on the structural parameters of a single hill of rice at each position. Second, according to the field planting configuration, including parameters such as row spacing, plant spacing, and the number of hills, the rice 3D structural models are positioned within the field scene. Finally, based on the optical properties of rice leaves, stems, and soil background, the reflectance and transmittance information for the corresponding structures are assigned to realize the construction of the rice 3D scene.

### 2.6 Sensitivity analysis of rice canopy structure to canopy spectra

To quantify the influence of rice canopy structural parameters and vertical leaf structural parameters on canopy spectral reflectance, this study utilized Random Forest (RF) to conduct a feature sensitivity analysis on canopy spectra. We established sensitivity analysis studies based on model input parameters for both the “Precision Mode” and the “Rapid Mode.” The “Precision Mode” analysis encompasses the importance of all parameters, the importance of different leaf positions, and the importance of different structural parameters, which are used to judge the impact of vertical leaf structural changes on canopy spectra from different dimensions. Since the analysis mainly focuses on the influence of the structural model on canopy spectra, the ridge characteristics of field rice were not considered. Based on the parameter ranges of measured data, 1000 rice 3D structures were simulated. Representative leaf spectra from measured data were selected, and the simulated 3D scene dimensions were set to 40 × 40 cm with one hill of rice placed in the center.

### 2.7 Rice canopy hyperspectral simulation method and verification

Considering that the rice 3D structural model constructed in this study is primarily intended for the rapid reconstruction of rice 3D scenes, simulating the effects of rice vertical leaf structure and spectral changes on rice canopy spectra, and the inversion of rice vertical leaf physicochemical parameters, it is necessary to verify the simulation accuracy of the rice 3D structural model for canopy spectra. This study plans to validate the accuracy of rice canopy reflectance simulation by integrating the LESS model with rice 3D scenes constructed under the two modes of the rice 3D structural model, supplemented by spectral simulation results based on the PROSAIL model for comparison. The LESS model is a commonly used 3D radiative transfer model in the remote sensing field, capable of achieving precise simulation of canopy reflectance by inputting 3D structural scene information. The PROSAIL model is a currently widely used one-dimensional radiative transfer model, which simulates canopy spectra by assuming the canopy to be a turbid homogeneous medium. Among the model input parameters, the leaf spectrum was set as the average value of all leaf spectra for each sample, while LAI, viewing geometry, leaf inclination distribution, and background spectra were kept consistent with the LESS model. We plan to evaluate model accuracy from two perspectives: full-waveband spectral error and common vegetation index error, and analyze the spectral simulation accuracy at different growth stages. The formulas for common vegetation indices are shown in Table S1.

### 2.8 Evaluation metrics

For the accuracy evaluation of leaf number in the rice vertical leaf structural parameter estimation method, this study primarily employs Accuracy, Precision, and Recall as evaluation metrics for model accuracy.

Accuracy represents the percentage of correctly predicted results out of the total samples, reflecting the overall prediction capability of the model. Precision denotes the probability of actual positive samples among all samples predicted as positive, measuring the proportion of correctly identified positive predictions. Recall indicates the probability of samples predicted as positive among actual positive samples, evaluating the model's ability to identify positive samples.

(10) $$\begin{array}{c} Accuracy=\frac{TP+TN}{TP+TN+FT+FN}\; \end{array}$$

(11) $$\begin{array}{c} Precision=\frac{TP}{TP+FP}\; \end{array}$$

(12) $$\begin{array}{c} Recall=\frac{TP}{TP+FN}\; \end{array}$$

For the estimation results of the remaining parameters and the canopy spectral simulation accuracy of the rice 3D structural model, this study mainly evaluates the accuracy using the Coefficient of Determination (R^2^) and the Root Mean Square Error (RMSE). The calculation methods are as follows:

(11) $$\begin{array}{c} R^{2}=\frac{\text{ESS}}{\text{TSS}}=1-\frac{\text{RSS}}{\text{TSS}\;}\; \end{array}$$

(12) $$\begin{array}{c} \text{RMSE}=\sqrt{\frac{\sum_{j=1}^{n}{\left(X_{\text{meas},j}-X_{\mathrm{mod},j}\right)}^{2}}{X_{\text{meas}}}} \end{array}$$

## 3 Results

### 3.1 Simulation results of rice 3D structure under “Precision Mode"

Based on the measurement methods for rice 3D structural parameters proposed in Section 2.2, the statistics of the acquired rice 3D structural parameters are presented in Table 1. Since the data acquisition spanned the jointing and booting stages and included four different nitrogen fertilizer gradients, the variation in rice growth was relatively significant, encompassing rice structural data with diverse parameter combinations.

**Table 1:** Statistical data of structural parameters.

Indices	Maximum values	Minimum values	Mean	Standard deviation
PH/cm	27.4000	113.000	66.7587	18.0828
LAI	1.2000	8.0300	4.4871	1.31960
AGB/g	1.4500	81.1800	19.4322	16.8237
Leaf Count	6.0000	2.0000	3.8100	0.8000
Stem Height/cm	75.4000	11.6000	42.3100	21.3000
Plant Count	40.0000	7.0000	22.6300	7.5100
Leaf Length/cm	16.2000	57.4000	30.0805	8.5020
Leaf width/cm	0.7000	2.0000	1.1442	0.2270
Leaf Position Ratio	0.2984	1.0000	0.8360	0.1937

### 3.2 Simulation results of rice 3D structure under “Rapid Mode"

Compared to the “Precision Mode,” the “Rapid Mode” aims to reduce the complexity of rice 3D structure generation. It selects four parameters—transplanting date, PH, LAI, and AGB—which can be rapidly acquired via UAV remote sensing inversion methods, as model inputs. By constructing estimation models for canopy and leaf structural parameters, estimated values of 3D structural parameters are obtained and used to construct the simulated rice 3D structure. Although the rice 3D models obtained through this method are subject to errors from the estimation models, resulting in lower accuracy compared to the “Precision Mode,” they possess significant advantages in terms of data acquisition cost and speed. In this section, this study utilizes three common machine learning models—SVM, RF, and XGBoost—to estimate rice structural parameters and evaluates the simulation accuracy of the 3D model under the “Rapid Mode” through a comparison of the results.

#### 3.2.1 Estimation results of rice canopy structural parameters

**Fig. 3:**
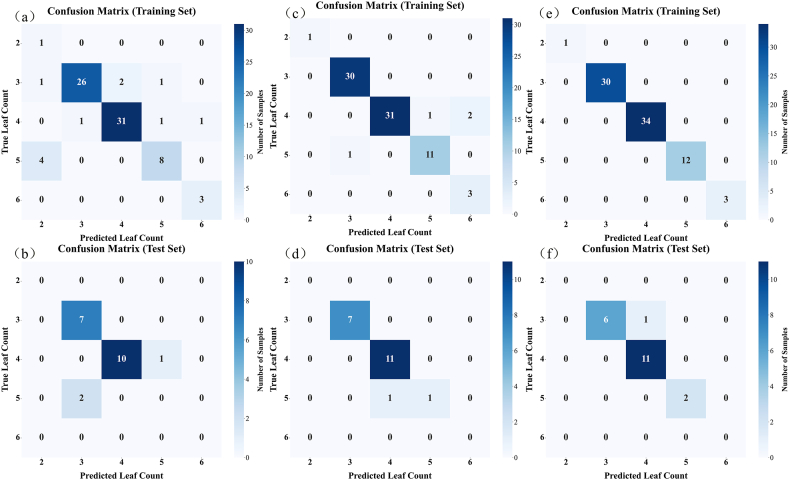
Confusion matrices for leaf number classification. (a) and (b) show the classification results for the SVM training and test sets; (c) and (d) for the RF training and test sets; and (e) and (f) for the XGBoost training and test sets.

**Fig. 4:**
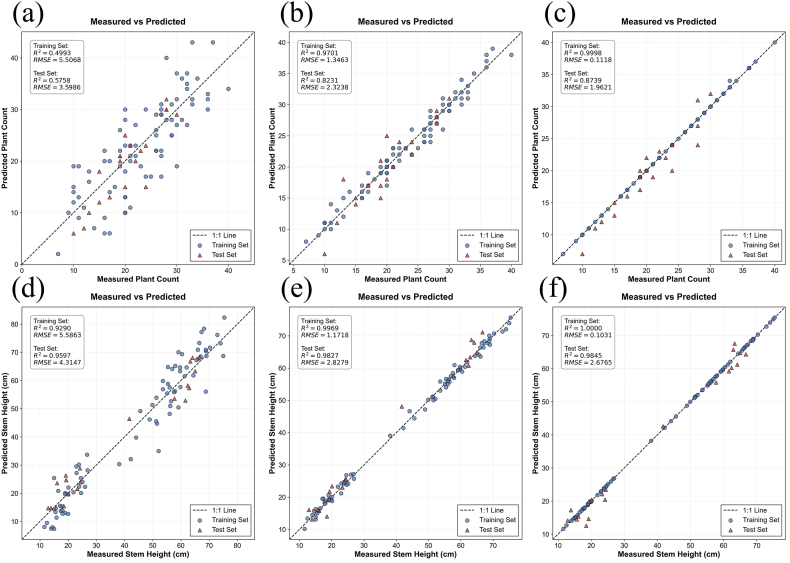
Estimation results of rice canopy structural parameters. (a), (b), and (c) display the estimation results for plant count using SVM, RF, and XGBoost models, respectively; (d), (e), and (f) display the estimation results for PH using SVM, RF, and XGBoost models, respectively.

This study employed three machine learning methods to estimate three rice canopy structural parameters: leaf number, tiller number, and stem height. Specifically, a classification method was adopted for leaf number, while regression methods were used for tiller number and stem height. The estimation results are presented in Table S2, Table S3, Fig. 3, and Fig. 4. It can be observed that, regarding the estimation methods, RF and XGBoost achieved higher overall estimation accuracy for canopy structural parameters. XGBoost performed slightly better than RF, while SVM performed relatively poorly.

Regarding different structural parameters, for leaf number, all three models maintained very high estimation accuracy, with accuracies of 1.00 and 0.9500 for the training and testing sets, respectively. For tiller number and stem height, the R^2^ of the training set was very high, approaching 1, while the R^2^ of the testing set was 0.8733 and 0.9845, respectively. The high estimation accuracy for stem height can be explained by the strong correlation between PH and stem height observed in the sensitivity analysis results. Considering that the development status of different tillers within a single hill of rice is relatively consistent, AGB and LAI can also explain the tiller number information to a certain extent. Overall, machine learning methods are capable of estimating canopy structural parameters with relatively high accuracy.

#### 3.2.2 Estimation results of rice vertical leaf structural parameters

**Fig. 5:**
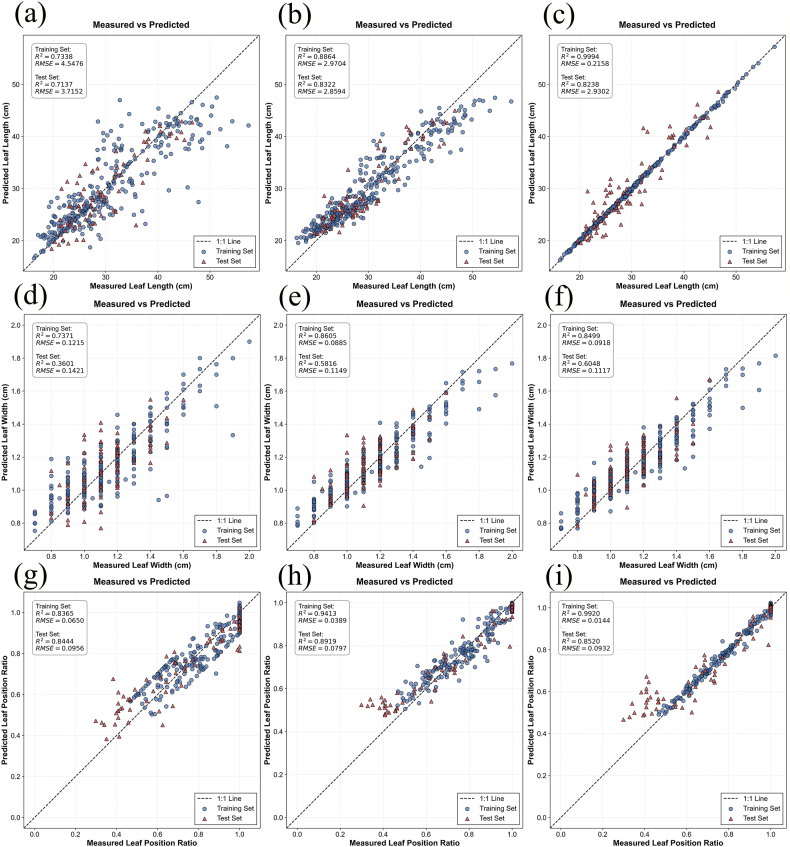
Estimation results of rice leaf structural parameters. (a), (b), and (c) display the estimation results for leaf length using SVM, RF, and XGBoost models, respectively; (d), (e), and (f) display the estimation results for leaf width using SVM, RF, and XGBoost models, respectively; (g), (h), and (i) display the estimation results for leaf position ratio using SVM, RF, and XGBoost models, respectively.

To estimate the vertical leaf structural parameters of rice, this study constructed an estimation model based on the predicted leaf number, incorporating leaf position as an input to predict leaf length, leaf width, and leaf position ratio at different leaf positions. The estimation results, as shown in Table S4 and Fig. 5, indicate that regarding the estimation methods—consistent with the canopy structural parameter results—the RF and XGBoost models achieved comparable accuracies, and both outperformed the SVM model. In terms of specific structural parameters, the RF model exhibited the best performance for leaf length, with a test set R^2^ of 0.8238 and an RMSE of 2.8594, while the XGBoost model showed lower accuracy and signs of overfitting. For leaf width, the accuracy of all three models was relatively insufficient with observed overfitting, though the XGBoost model performed the best with a test set R^2^ of 0.6048 and an RMSE of 0.1117. For the leaf position ratio, the accuracies of the three models were comparable, with the RF model achieving the highest accuracy (R^2^ = 0.8919, RMSE = 0.0897). Overall, while the models guaranteed high estimation accuracy for leaf length and leaf position ratio, the accuracy for leaf width was relatively poor. This discrepancy may be attributed to the fact that while regularities related to leaf position exist within a single plant, the mixed modeling of all leaf data hinders the capture of interactions between structural parameters within individual plants; furthermore, actual rice growth is influenced by factors such as nutritional status and individual variability, and parameters such as LAI and AGB have a relatively weak correlation with leaf width.

### 3.3 Sensitivity analysis of rice canopy structure on canopy spectra

To analyze the influence of rice canopy structure and vertical leaf structure on spectral reflectance, this study employed the RF feature importance analysis method to assess the sensitivity of different canopy structural parameters under two scenarios. Since plant count is a critical variable affecting parameters such as canopy LAI and AGB, and thus exhibits a strong influence on canopy spectra, the importance analysis was conducted in two parts: one including plant count and one excluding it.

**Fig. 6:**
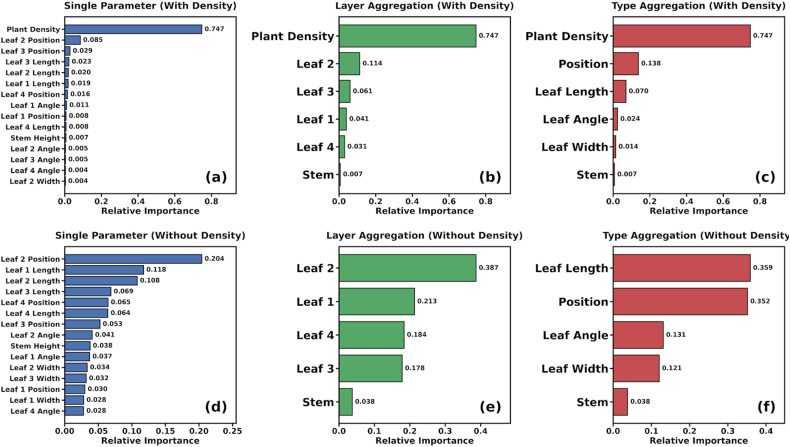
Sensitivity analysis results of rice structural parameters.

The results, shown in Fig. 6, indicate that whether viewed from the perspective of leaf layers or specific parameters, the influence of plant count on canopy spectra far exceeds that of other parameters, accounting for 74.7% of the total importance. This is theoretically consistent with the significant impact of planting density on canopy spectra, as higher density increases the probability of multiple scattering within the canopy.

To better analyze the importance of vertical leaf structure on canopy spectra, the structural parameters were ranked after excluding the plant count variable. The results demonstrate that, in terms of leaf layers, the 2ndleaf from the top (L2) showed the highest importance, reaching 38.7%, which is significantly higher than the 21.3% observed for the flag leaf (L1). This difference arises because although L1 is at the top, it is more erect and has a smaller leaf area; in contrast, L2 is more spread out and accounts for a larger proportion of the effective canopy leaf area. Meanwhile, the importance of the 3rd (L3) and 4th (L4) leaves from the top was relatively similar, accounting for 17.8% and 18.4%, respectively. This similarity is due to the shading of lower leaves by the upper layers. Furthermore, within the row structure of rice, the increased mutual shading effect between different plants may lead to a further reduction in their contribution ratios.

From the perspective of morphological parameters, leaf length and leaf position showed similar importance, at 35.9% and 35.2%, respectively, which was significantly higher than that of leaf inclination angle and leaf width. This is because leaf length contributes more significantly to leaf area, while leaf position primarily affects canopy gap fraction; consequently, the importance of leaf width and leaf inclination angle is relatively lower.

Regarding the overall parameters, L2 exhibited the highest importance, followed by the leaf length of L1 and L2, while the importance of the remaining parameters was relatively minor. Notably, the importance of PH was far lower than that of many leaf parameters, reflecting that the stem is heavily shaded by leaves and its variation has a minimal impact on canopy spectra.

### 3.4 Simulation results of rice canopy hyperspectral reflectance under two modes

**Fig. 7:**
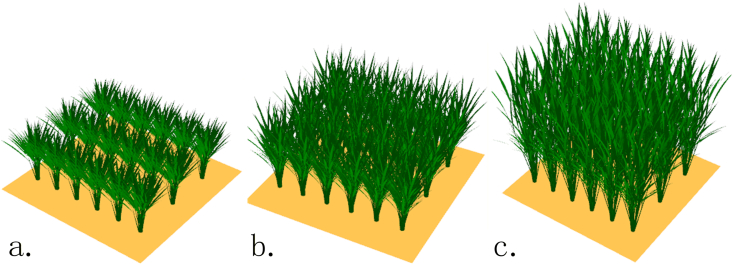
Simulation results of rice 3D scenes. (a), (b), and (c) display the simulation results for the tillering, jointing, and booting stages, respectively.

To investigate the differences in simulation accuracy of canopy hyperspectral reflectance between the two rice 3D structural modeling modes, this study generated 3D rice structures using the “Precision Mode” and “Rapid Mode,” respectively. These structures were combined with actual rice planting density, leaf spectral reflectance at different leaf positions, and soil background reflectance to construct 3D rice scenes. The scene area was set to1m∗1m, consisting of 3 rows with 6 hills per row. The planting density was defined by a row spacing of 30 cm and a plant spacing of 15 cm. The generated 3D scenes are shown in Fig. 7.

#### 3.4.1 Simulation of rice canopy reflectance

**Fig. 8:**
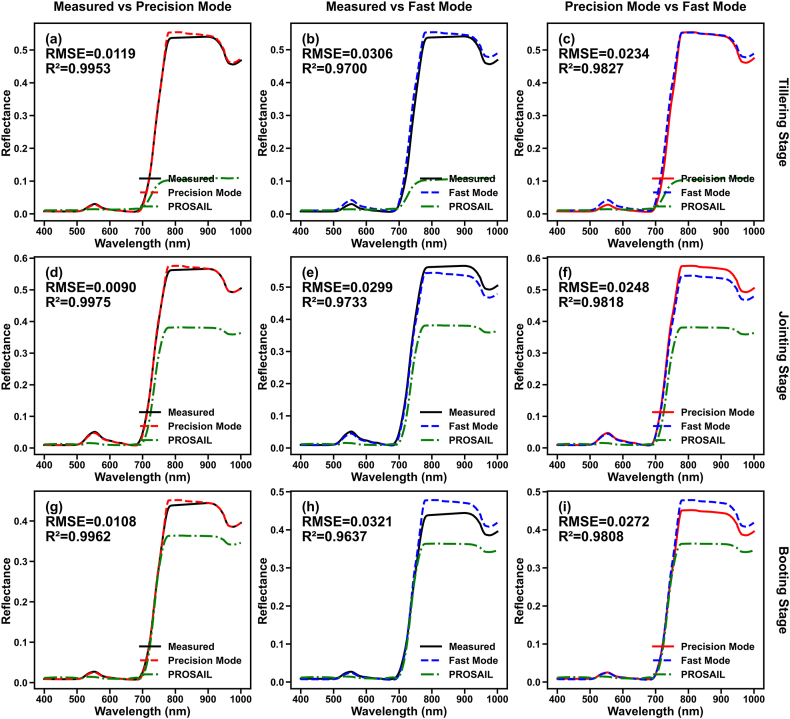
Simulation results of rice canopy spectra at different growth stages. (a), (b), and (c) show the comparison of three types of spectra at the tillering stage; (d), (e), and (f) show the comparison at the jointing stage; and (g), (h), and (i) show the simulation results at the booting stage.

By combining the simulated rice 3D scenes generated under the two modes with the LESS model, rice canopy reflectance was simulated and compared with UAV-acquired canopy reflectance. Additionally, the simulation accuracy of several common vegetation indices was calculated. The hyperspectral simulation results are presented in Table S5 and Fig. 8.

Overall, compared to the PROSAIL model, both the “Precision Mode” and “Rapid Mode” effectively simulated rice canopy spectra. However, the simulation results of the Precision Mode were significantly superior to those of the Rapid Mode, achieving average RMSE and R^2^ values of 0.0104 and 0.9965, respectively, whereas the Rapid Mode achieved an accuracy of 0.0307 and 0.9694. This indicates that although the Rapid Mode reduces sampling effort and improves modeling efficiency, estimation errors in rice structural parameters still impact the simulation accuracy of canopy spectra.

From the perspective of different growth stages, the performance of the two methods varied slightly. The spectra obtained from the Precision Mode showed the lowest accuracy at the tillering stage and the highest at the jointing stage. This discrepancy may be attributed to the larger volume of sampling data available at the jointing stage, causing the leaf inclination angle function to be biased towards the distribution characteristics of that stage; meanwhile, rice leaves at the tillering stage are more erect, resulting in actual leaf inclination angles that are higher than the values derived from the function. Conversely, the Rapid Mode spectra exhibited the lowest accuracy at the booting stage, with comparable accuracy between the tillering and jointing stages. This likely stems from the physiological transition of rice from vegetative growth to reproductive growth during the booting stage, leading to changes in vertical leaf structural patterns that the model failed to fully capture. Furthermore, the simulation accuracy of the PROSAIL model gradually improved with rice development; this is likely due to the increase in canopy density as the rice matures, which aligns more closely with the uniform canopy turbid medium assumption of the PROSAIL model.

#### 3.4.2 Simulation accuracy of vegetation indices

The simulation accuracy of common vegetation indices is presented in Table S7. It can be observed that the “Precision Mode” achieved relatively high simulation accuracy for vegetation indices, although the accuracy for CIrededge was lower. This lower accuracy is attributed to the high slope of the simulated spectra in the red-edge region. Conversely, the “Rapid Mode” exhibited relatively larger simulation errors for vegetation indices. For NDVI, although the simulation RMSE was small, the R^2^ was low. This may be because the high planting density of rice caused NDVI to approach saturation, thereby amplifying the perturbations caused by estimation errors in leaf structural parameters.

## 4 Discussion

### 4.1 Analysis of rice 3D scene construction methods

3D radiative transfer models, which simulate canopy spectra based on canopy 3D structure and leaf spectral data, represent a frontier in rice remote sensing modeling. Given the complexity of the rice canopy, these models are superior to 1D models like PROSAIL in simulating the influence of canopy structure on spectral reflectance [10]. However, since the simulation accuracy of 3D radiative transfer models depends on the fidelity of the canopy structure reconstruction, the rapid and precise acquisition of rice canopy structural information remains a critical bottleneck for practical applications. Current research primarily relies on point cloud data obtained via LiDAR or oblique photography, employing point cloud reconstruction methods to approximate canopy structures [24]. Some studies have also attempted to integrate plant 3D structural models to achieve precise simulations [25].

Nevertheless, 3D structures derived from surface mesh methods often lack a detailed representation of rice physiological structures, making it difficult to reflect the impact of vertical heterogeneity—specifically, the structure and spectra of leaves at different positions—on canopy spectra. While some rice 3D structural models exist, they often prioritize growth and phenotype research [26], resulting in low efficiency and poor compatibility when applied to radiative transfer simulations. Furthermore, compared to terrestrial laser scanning, UAV-based point cloud data typically suffer from lower density and noise interference, hindering their integration with existing structural models and limiting the application of 3D radiative transfer models in vegetation parameter estimation. To address these issues, this study developed a rice 3D structural model that can be rapidly constructed based on typical canopy structural parameters. This approach aims to support analyses of the impact of vertical heterogeneity on canopy spectra, the retrieval of nutritional parameters, and the estimation of photosynthetically active radiation (PAR) using 3D radiative transfer models.

Precise simulation of the rice 3D structure requires parameters including PH, plant count, and the leaf length, width, inclination angle, and position ratio for each leaf. However, UAV point cloud data struggle to meet the precision requirements for estimating all these parameters. Therefore, based on an analysis of measured data and considering representativeness, acquisition difficulty, and estimation accuracy from UAV point cloud data in current literature, this study selected transplanting date, PH, AGB, and LAI as model inputs. Numerous studies have demonstrated that PH, AGB, and LAI can be estimated with high accuracy using UAV remote sensing data [27,28]. Moreover, these three parameters are not only common indicators of rice growth status but also key inputs for many crop growth models, 3D structural models, and radiative transfer models [29], facilitating future model expansion. To derive specific 3D structural parameters from these four inputs, this study employed three machine learning methods—SVM, RF, and XGBoost. The results indicated that parameters including leaf number, plant count, PH, leaf length, and leaf position ratio were estimated with good accuracy. These are critical parameters affecting canopy spectra. However, the estimation accuracy for leaf width was relatively lower, likely due to errors introduced by sampling data from the early jointing-booting stage, where upper leaves were not fully developed. Future work should optimize the estimation model by incorporating leaf inclination distribution functions and other rice growth laws.

### 4.2 Analysis of rice canopy spectral simulation

Rice canopy spectral simulation currently relies primarily on 1D radiative transfer models represented by PROSAIL and 3D models represented by LESS and DART [30]. At the leaf scale, both model types typically use PROSPECT or its extensions. At the canopy scale, PROSAIL describes the canopy as a turbid medium defined by LAI and a leaf inclination distribution function. Compared to 3D models, the canopy assumptions in PROSAIL struggle to detail the row structure and vertical leaf structure of rice. Although some studies have optimized PROSAIL by assuming a multi-layer heterogeneous structure, it remains difficult to describe heterogeneity in the horizontal dimension. In contrast, 3D radiative transfer models, combined with ray-tracing methods, can effectively describe the impact of structural and spectral vertical heterogeneity on canopy spectra based on realistic 3D scenes [31], making them essential for studying fine-scale structural effects. Therefore, this study constructed a rice structural simulation model using vertical leaf structural parameters as inputs and validated its spectral simulation accuracy using the LESS model. The results showed that the “Precision Mode” effectively simulated canopy spectra and vegetation indices, with an average RMSE of 0.0104 and an R2 of 0.9965, indicating that the model well reflects the influence of structural parameters on spectral response.

In practical research, it is common to estimate leaf physicochemical parameters using easily accessible canopy spectral data. Models based on hybrid modeling methods typically possess good transferability and robustness. However, accurate estimation—especially for vertical distribution of physicochemical parameters—requires decoupling the influence of canopy structure on spectra [32,33]. While many studies have focused on retrieving canopy structural parameters like LAI, AGB, and PH from UAV data, the precision of UAV data often limits the direct acquisition of internal vertical structural information. Therefore, this study developed a “Rapid Mode” based on the “Precision Mode,” using canopy structural parameters as inputs. Although the “Rapid Mode” uses machine learning to estimate vertical leaf structural parameters, leading to less stable accuracy and potential error propagation from LAI and AGB estimation errors, its efficiency in constructing 3D scenes is significantly superior to the “Precision Mode.” Crucially, it is compatible with hybrid models based on UAV data. Validation results showed that while the “Rapid Mode” lagged behind the “Precision Mode” in spectral simulation accuracy—particularly for NDVI under saturation effects (average RMSE = 0.0307, R^2^ = 0.9694)—it still possesses unique application scenarios and value.

### 4.3 Limitations and future prospects

Overall, although the current rice 3D structural model can rapidly generate rice vertical-scale 3D structures based on canopy structural parameters and effectively simulate the influence of rice vertical-scale leaf spectra on canopy spectra, several issues remain to be addressed. First, the current model is primarily based on a single rice variety without considering variety differences. Although this approach may improve the estimation accuracy of rice vertical structure, it also results in insufficient model generalization capability. Different rice varieties exhibit variations in developmental processes and structural characteristics. Quantifying these structural differences based on limited field measurements and incorporating transfer learning methods to enhance model generalization may represent a viable solution. Second, the model relies on PH, LAI, and AGB estimated through remote sensing methods to estimate 3D structural parameters, which may lead to error propagation. Although current approaches using UAV-based hyperspectral imagery or LiDAR can estimate these three parameters with relatively high accuracy, the resulting errors still affect the simulation accuracy of canopy structure. Considering that the importance analysis indicates that plant density has an influence of 0.747 on canopy spectra, directly estimating plant density through remote sensing modeling methods may mitigate the impact of error propagation on spectral simulation accuracy. Third, the model does not incorporate a rice panicle model, and due to the current lack of effective simulation methods for panicle and stem spectra, the model employs mean leaf spectra for stems. Although rice canopy spectra are primarily influenced by leaf spectra and structure, with minor contributions from panicles and stems, neglecting these components may still affect canopy spectral simulation accuracy.

Despite these limitations, the ability to rapidly generate rice canopy 3D structures considering vertical leaf differences using only a few parameters has significant implications. For instance, simulated data from the model can be used to investigate the impact of structural differences at various vertical levels on canopy spectra, including the contribution of leaves at different positions. Alternatively, it can facilitate the rapid generation of 3D scenes based on hyperspectral or LiDAR data, supporting hybrid modeling for nutritional parameter retrieval using LESS. Furthermore, since the main input parameters can be obtained from models like WOFOST and D-SART, this model can serve as a vital tool bridging crop growth models and 3D radiative transfer models, enabling time-series canopy spectral simulation and PAR estimation. In summary, the rice 3D structural model constructed in this study offers an effective method for analyzing vertical structural effects and rapidly constructing 3D scenes, holding significant value for radiative transfer modeling and nutritional parameter estimation.

## 5 Conclusion

To address the challenges of complex vertical canopy structures and low efficiency in 3D radiative transfer simulations in rice remote sensing, this study constructed a parameterized rice 3D structural model and explored rapid parameter acquisition via machine learning and its spectral simulation accuracy. The main conclusions are as follows:

(1) A rice 3D structural model capable of characterizing vertical heterogeneity was constructed. This study adopted a parameterized modeling method based on vertical structural parameters. This method not only realistically reconstructs plant type and population structure but also flexibly simulates 3D scenes for different growth stages and varieties by adjusting parameters, effectively overcoming the inability of models like PROSAIL to describe vertical heterogeneity.
(2) The high fidelity of the “Precision Mode” in spectral simulation was validated. Importing 3D scenes constructed from measured structural parameters into the LESS model for spectral simulation yielded high-precision canopy reflectance data in both visible and near-infrared bands. Comparison with measured spectra showed an RMSE of only 0.0104 and an R^2^ of 0.9965, proving the reliability of this 3D geometric model for radiative transfer simulation and its potential as a substitute for “ground truth” in mechanistic studies.
(3) An application-oriented “Rapid Mode” was developed and its applicability evaluated. Addressing the difficulty of acquiring fine-scale parameters in practice, this study selected four easily accessible parameters—PH, AGB, LAI, and transplanting date—as inputs and compared the ability of RF, SVM, and XGBoost algorithms to deduce fine structural parameters. The results indicated that while the XGBoost-based “Rapid Mode” was slightly inferior to the “Precision Mode” in accuracy, it drastically reduced data acquisition thresholds and modeling time. This mode maintains relatively high spectral simulation accuracy while significantly improving the operability of 3D simulation for large-area UAV monitoring, laying a foundation for future vertical retrieval of rice physicochemical parameters based on hybrid models.

## Author contributions

BJ, YF, and WC designed the experiment and performed the final critical review. BJ, TY, HY, and WR performed experiments and data analysis. BJ and YF wrote the manuscript. YF and WC provided critical review.

## Funding

This research was funded by the Platform Project of the Education Department of Liaoning Province (Grant No. JYTPT2024002).

## Declaration of competing interest

The authors declare that they have no known competing financial interests or personal relationships that could have appeared to influence the work reported in this paper.

## Data Availability

The data collected and used in this study are publicly available at: https://github.com/baijc4095-code/2024data. The code used for analysis can be obtained from the corresponding author upon reasonable request.
