# Supplementary material for: Rapid modeling of 3D rice canopy structure considering vertical heterogeneity and analysis of spectral response
Source: Plant Phenomics. 2026 May 25;8(3):100226. doi: 10.1016/j.plaphe.2026.100226 (PMC13235349; doi:10.1016/j.plaphe.2026.100226)
Supplement: Multimedia component 1 [file mmc1.docx]

**Supplementary Materials**


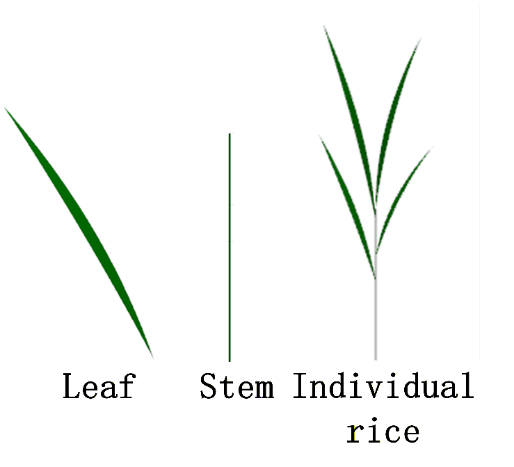


Figure S1. Models of leaf, stem, and single rice plant


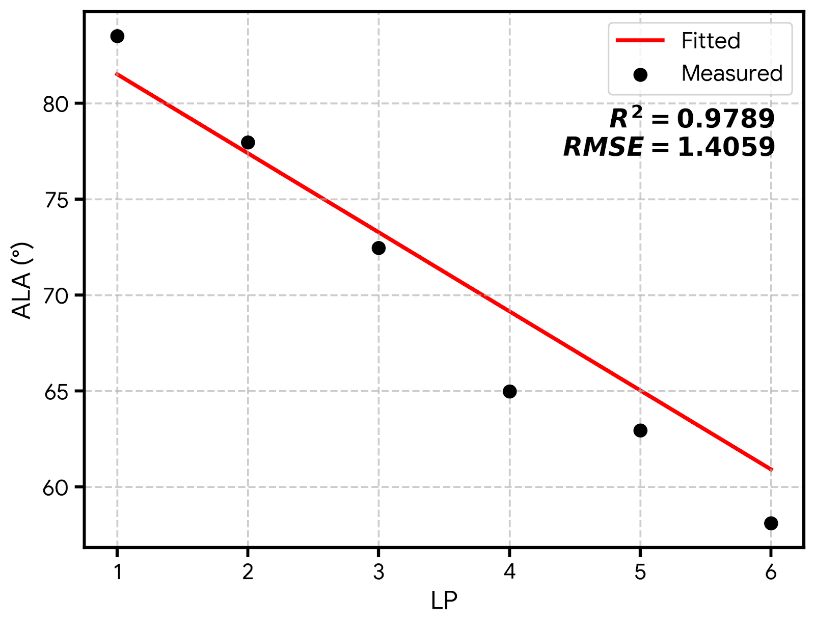


Figure S2. Fitting results of leaf inclination angle function


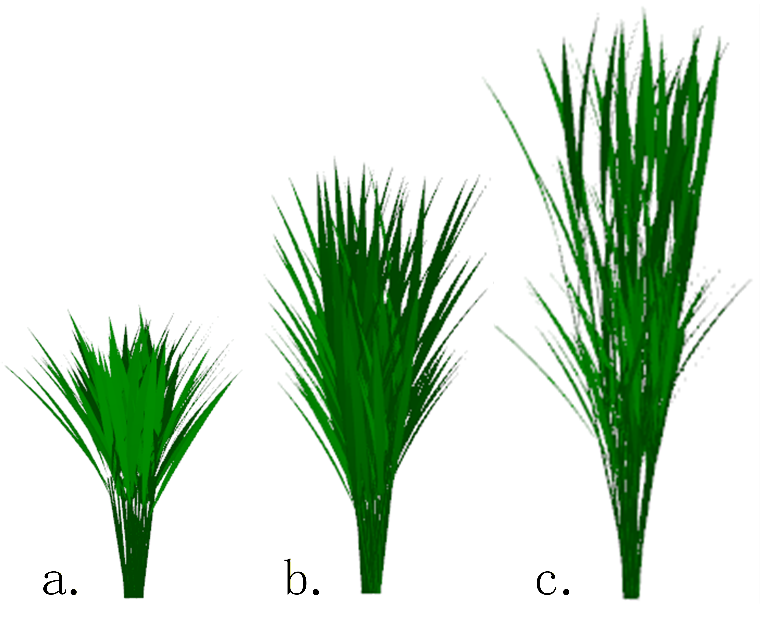


Figure S3. Simulation results of rice 3D structure, where (a), (b), and (c) represent the simulation results for the tillering stage, jointing stage, and booting stage, respectively.


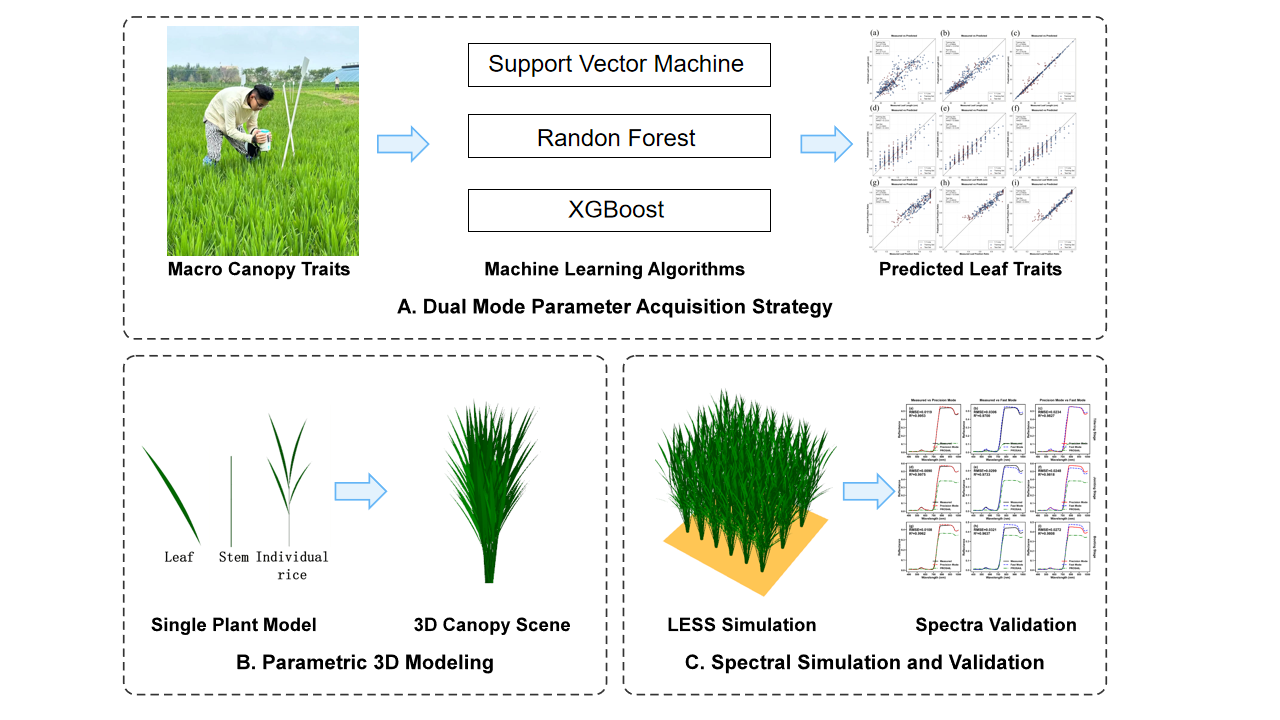


Figure S4. Technology Roadmap

Table S1. Formulas of common vegetation indices

| Index | Formula |
| --- | --- |
| NDVI | (NIR−Red)/(NIR+Red) |
| GNDVI | (NIR−Green)/(NIR+Green) |
| NDRE | (NIR−RedEdge)/(NIR+RedEdge) |
| CIrededge | (NIR/RedEdge)−1 |
| CIgreen | (NIR/Green)−1 |

Table S2. Classification results of leaf number

| Structural parameter | Method | Accuracy Train | Accuracy Test | Accuracy Test | Precision Train | Recall Train | Precision Test |
| --- | --- | --- | --- | --- | --- | --- | --- |
| Leaf Count | SVM | 0.8625 | 0.8500 | 0.85 | 0.7238 | 0.889 | 0.5926 |
|  | RF | 0.9500 | 0.9500 | 0.95 | 0.8969 | 0.9657 | 0.9722 |
|  | XGBOOST | 1.0000 | 0.9500 | 0.95 | 1 | 1 | 0.9722 |

Table S3. Estimation results of plant count and PH

| Structural parameter | Method | Train  R^2^ | Train RMSE | Test  R^2^ | Test RMSE |
| --- | --- | --- | --- | --- | --- |
| Plant Count | SVM | 0.4993 | 5.5068 | 0.5758 | 3.5986 |
|  | RF | 0.9701 | 1.3463 | 0.8231 | 2.3238 |
|  | XGBOOST | 0.9998 | 0.1118 | 0.8739 | 1.9621 |
| Stem Height(cm) | SVM | 0.9290 | 5.5863 | 0.9597 | 4.3147 |
|  | RF | 0.9969 | 1.1718 | 0.9827 | 2.8279 |
|  | XGBOOST | 1.0000 | 0.1031 | 0.9845 | 2.6765 |

Table S4. Estimation results of leaf structural parameters.

| Structural parameter | Method | Train R^2^ | Train RMSE | Test R^2^ | Test RMSE |
| --- | --- | --- | --- | --- | --- |
| Leaf Length(cm) | SVM | 0.7338 | 4.5476 | 0.7137 | 3.7352 |
|  | RF | 0.8864 | 2.9704 | 0.8322 | 2.8594 |
|  | XGBOOST | 0.9994 | 0.2158 | 0.8238 | 2.9302 |
| Leaf Width(cm) | SVM | 0.7371 | 0.1215 | 0.3601 | 0.1421 |
|  | RF | 0.8605 | 0.0885 | 0.5816 | 0.1149 |
|  | XGBOOST | 0.8499 | 0.0918 | 0.6048 | 0.1117 |
| Leaf Position Ratio | SVM | 0.8365 | 0.0650 | 0.8444 | 0.0956 |
|  | RF | 0.9413 | 0.0389 | 0.8919 | 0.0797 |
|  | XGBOOST | 0.9920 | 0.0144 | 0.8520 | 0.0932 |

Table S5. Simulation results of rice canopy spectra under different modes and growth stages.

| Metric | Tillering | Jointing | Booting | All stages |
| --- | --- | --- | --- | --- |
| Measured-Precise RMSE | 0.0119 | 0.009 | 0.0108 | 0.0104 |
| Measured-Precise R^2^ | 0.9953 | 0.9975 | 0.9962 | 0.9965 |
| Measured-Fast RMSE | 0.0306 | 0.0299 | 0.0321 | 0.0307 |
| Measured-Fast R^2^ | 0.9700 | 0.9733 | 0.9637 | 0.9694 |
| Precise-Fast RMSE | 0.0234 | 0.0248 | 0.0272 | 0.0251 |
| Precise-Fast R^2^ | 0.9827 | 0.9818 | 0.9776 | 0.9808 |
| Measured-PROSAIL RMSE | 0.1089 | 0.0600 | 0.0410 | 0.0747 |
| Measured-PROSAIL R^2^ | -14.9880 | -2.6534 | -1.3755 | -4.2921 |

Table S6. Sampling Time and Sample Quantity

| Growth Stage | Sampling Time | Sample Quantity | Retained Quantity |
| --- | --- | --- | --- |
| Tillering Stage | 2024.6.30 | 16 | 12 |
|  | 2024.7.4 | 16 | 13 |
| Jointing Stage | 2024.7.9 | 16 | 14 |
|  | 2024.7.12 | 16 | 15 |
|  | 2024.7.18 | 16 | 15 |
| Booting Stage | 2024.7.23 | 16 | 16 |
|  | 2024.8.6 | 16 | 15 |

Table S7. Simulation results of rice vegetation indices based on different modes

| Metric | NDVI | GNDVI | NDRE | CIrededge | CIgreen |
| --- | --- | --- | --- | --- | --- |
| Measured-Precise RMSE | 0.0031 | 0.0159 | 0.0220 | 1.6021 | 1.5871 |
| Measured-Precise R^2^ | 0.8431 | 0.9122 | 0.8628 | 0.6440 | 0.8621 |
| Measured-Fast RMSE | 0.0059 | 0.0241 | 0.0290 | 1.7537 | 2.295 |
| Measured-Fast R^2^ | 0.4353 | 0.7966 | 0.7611 | 0.5734 | 0.7116 |
| Precise-Fast RMSE | 0.0061 | 0.0239 | 0.0285 | 1.8039 | 2.5909 |
| Precise-Fast R^2^ | 0.1608 | 0.7622 | 0.7736 | 0.7214 | 0.7047 |
| Measured-PROSAIL RMSE | 0.0431 | 0.0811 | 0.0824 | 6.6324 | 12.9161 |
| Measured-PROSAIL R^2^ | -28.8776 | -1.2954 | -0.9290 | -5.1009 | -8.1324 |
